# Identity-by-descent refines mapping of candidate regions for preaxial polydactyly II /III in a large Chinese pedigree

**DOI:** 10.1186/s41065-017-0040-6

**Published:** 2017-07-03

**Authors:** Xingyan Yang, Quankuan Shen, Xierzhatijiang Sulaiman, Hequn Liu, Minsheng Peng, Yaping Zhang

**Affiliations:** 1 State Key Laboratory for Conservation and Utilization of Bio-resources in Yunnan, Yunnan University, Kunming, China; 2 State Key Laboratory of Genetic Resources and Evolution, Kunming Institute of Zoology, Kunming, China; 3 Kunming College of Life Science, University of Chinese Academy of Sciences, Kunming, China; 4 KIZ /CUHK Joint Laboratory of Bio-resources and Molecular Research in Common Diseases, Kunming, China; 5 Basic Medical College, Xinjiang Medical University, Ürümqi, 830011 China; 6 Kunming Institute of Zoology, Chinese Academy of Sciences, 32 Jiaochang Donglu, Kunming, 650223 China

**Keywords:** PPD, IBD, 7q36, *LMBR1*, *SHH*

## Abstract

Preaxial polydactyly (PPD) is congenital hand malformation characterized by the duplication of digit. Herein, we scan the genome-wide SNPs for a large Chinese family with PPD-II/III. We employ the refined IBD algorithm to identify the identity-by-decent (IBD) segments and compare the frequency among the patients and normal relatives. A total of 72 markers of 0.01 percentile of the permutation are identified as the peak signals. Among of them, 57markers locate on chromosome 7q36 which is associated with PPD. Further analyses refine the mapping of candidate region in chromosome 7q36 into two 380 Kb fragments within *LMBR1* and *SHH* respectively. IBD approach is a suitable method for mapping causal gene of human disease. Target-enrichment sequencing as well as functional experiments are required to illustrate the pathogenic mechanisms for PPD in the future.

**Electronic supplementary material:**

The online version of this article (doi:10.1186/s41065-017-0040-6) contains supplementary material, which is available to authorized users.

## Main text

### Background

Preaxial polydactyly (PPD; OMIM#188740) is characterized as complete or partial duplication of the thumb [1]. It is one of the most common congenital deformities [2]. The worldwide incidence of PPD is 1 in 3000 births [3]. The prevalence rate of polydactyly in Chinese ranks third in birth defects after congenital heart diseases and central nervous system diseases [4]. Polydactyly has genetic and clinical heterogeneity [2]. The mainstream treatment is resection for excess digits.

A series of efforts have been performed to investigate the genetic basis for PPD. Zguricas et al. conducted linkage analysis for Dutch, British, Turkish, Cuban pedigrees and mapped the candidate region to a 1.9 cM interval between D7S550 and D7S2423 of 7q36 region [5]. Heus et al. further refined the candidate region to approximately 450 Kb including five genes: *C7orf2* (i.e. *LMBR1*), *C7orf3* (i.e. *NOM1*), *C7orf4* (i.e. *LINC00244*), *HLXB9* (i.e. *MNX1*) and *RNF32* [6] by reconstructed a detailed physical map using a combination of exon trapping, cDNA selection, and EST mapping methods. Further evidence shows that PPD is caused by ectopic expression of *SHH* in mice, cats and humans [7]. The zone of polarizing activity regulatory sequence (ZRS), performs as the limb-specific cis-regulator, in controlling the expression of *SHH*. ZRS locates within intron 5 of the neighboring gene *LMBR1*, which is ~1 Mb upstream from *SHH* [8]. In a number of cases, mutations of ZRS disturb the expression of *SHH* at the anterior limb bud margin and consequently caused PPD [8–15]. Homozygous deletion of ZRS can cause limb-specific absence of *SHH* expression in the acheiropodia [16]. It actually exists in the snake species and a limbless newt [17]. Duplication of ZRS results in Triphalangeal thumb–polysyndactyly syndrome (TPTPS; OMIM#174500), that is a subtype of PPD. It also can lead to syndactyly type IV (SD4; OMIM#186200) [18].

The common PPD only involves in hands/feet. In extreme and rare cases, PPD occur both in hands and feet. To investigate the genetic basis, Li et al. adopted a candidate gene approach to genotype nine microsatellite markers of 7q36 chromosomal region in a Chinese family with PPD both in hands and feet. By linkage analysis and haplotype construction, they located the linked region spanning 1.7 Mb between D7S2465 and D7D2423 [19]. It includes the 450 kb candidate region previously identified by Henus [6]. Nevertheless, the other parts of genome is not investigated yet. Herein, we genotyped genome-wide SNPs and employed the identity-by-descent (IBD) to refine the mapping of potential candidate loci for PPD in the same family.

## Methods

### Patients

This study has been approved by the internal review board of Kunming Institute of Zoology, Chinese Academy of Sciences (SMKX 2012013). The six-generation pedigree (including 21 patients and 24 normal relatives) involved in this study has been described previously in Li et al. [19]. All patients show hexadactyly of hands and feet. They have been diagnosed by physical examination & X-ray and assigned as isolated PPD-II on hand and isolated PPD-III on feet according to Temtamy and McKusick’s classification [20]. PPD shows autosomal dominant inheritance in this pedigree.

### SNP array

We genotyped 900,015 markers in 45 individual with HumanOmniZhongHua-8 BeadChip v1.0 (Illumina). We exported the chip data in accordance with the reference sequence GRCh37 into PLINK format via GenomeStudio (Illumina). The markers on mitochondrial DNA and sex chromosomes were disregarded. We adopted a series of quality control strategies [21] by using PLINK 1.9 [22]. Two individuals with call rate < 90% were removed. The SNPs with call rate < 90%, minor allele frequency < 1%, and deviation of Hardy–Weinberg equilibrium (*P* < 1e-6) were excluded. After filtering, a total of 595,534 autosomal SNPs for 43 individuals were utilized in subsequent analyses. The data have been deposited into the Genome Variation Map [23] (GVM000001).

### IBD detection

We used BEAGLE 4.0 [24] to phase and impute the genotype data referring to the pedigree information and the genetic map of HapMapII [25]. We detected the IBD segment with the refined IBD in BEAGLE 4.1 [26]. The IBD segment length shorter than 1 cM and the logarithm of odds (LOD) score under 3 were excluded before permutation [27]. The threshold of the genome-wide significance was set to the 0.05 percentile of the distribution of the permutation *p*-value.

## Results

**Fig. 1:**
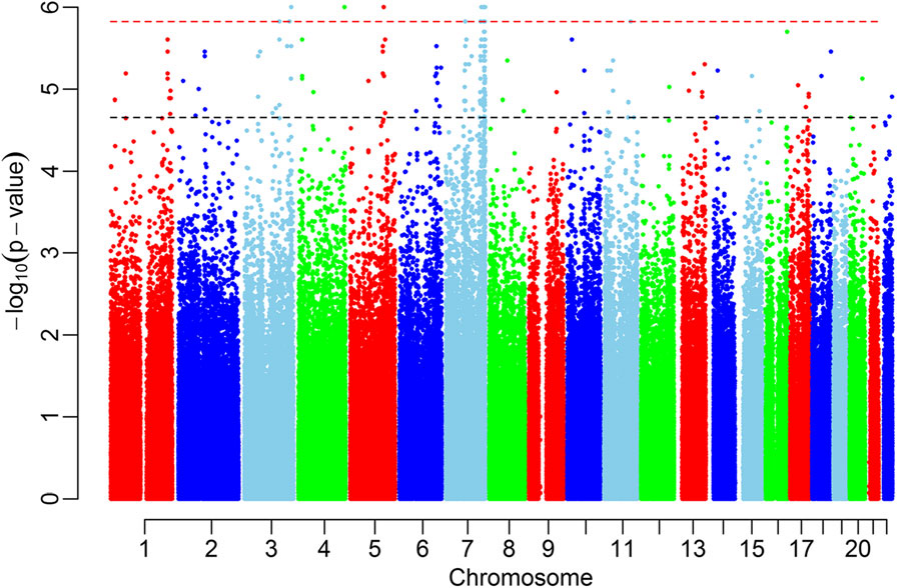
Permutation analysis after filtering out regions with low IBD sharing. The black line indicates genome-wide threshold and the red line is the 0.01 percentile of the permutation

The length distribution of detected IBD segments approximates a Pareto distribution (Additional file 1: Figure S1). The permutation result shows the significant segments distributing widely across genomes (Fig. 1). When considering the top 0.01% outliers of signals, we find the peak signals of 72 SNPs, of which 57 markers locate at 7q36 chromosomal region (Additional file 2: Table S1). We map the markers into the IBD fragments including *LMBR1* and *SHH* (Table 1). The minimal IBD segments within *LMBR1* and *SHH* are around 380 Kb, respectively (Additional file 3: Table S2). The IBD segments are more frequently in patient-patient (ratio; percentage) than normal-normal (ratio; percentage) (Table 2). We make annotation for the significant SNPs (Additional file 2: Table S1). All the SNPs are not haven’t been reported to be associated with PPD before.

**Table 1:** Genetic variants in the two IBD segments

Gene(7q36))	Position (GRCH37.p13)	SNP ID	REF	ALT	P-valve	Note
*LMBR1 156470537...156685902*	156354434	rs1860156	T	C	1.00E-06	116 kb upstream of *LMBR1*
	156401455	kgp6282999	C	A	1.00E-06	69 kb upstream of *LMBR1*
	156477347	kgp13575466	C	A	1.00E-06	
	156497668	rs10228997	A	G	1.00E-06	
	156526645	rs10224728	T	G	1.00E-06	
	156686101	kgp6457815	C	T	1.00E-06	199 bp downstream of *LMBR1*
	156687282	kgp1716770	C	T	1.00E-06	1 kb downstream of *LMBR1*
	156716316	kgp3747986	T	C	1.00E-06	30 kb downstream of *LMBR1*
	156730688	kgp7566181	T	C	1.00E-06	45 kb downstream of *LMBR1*
*SHH 155595558...155604967*	155103781	rs13223383	G	T	1.00E-06	492 kb upstream of *SHH*
	155,169,143	rs1990808	C	T	1.00E-06	426 kb upstream of *SHH*
	155,182,442	kgp9710825	G	A	1.00E-06	426 kb upstream of *SHH*
	155716520	rs4716928	C	T	1.00E-06	112 kb downstream of *SHH*
	155718241	rs4716930	A	C	1.00E-06	113 kb downstream of *SHH*
	155721324	rs11764820	A	G	1.00E-06	116 kb downstream of *SHH*
	155721386	rs11769663	G	T	1.00E-06	116 kb downstream of *SHH*
	155722231	rs6971588	T	G	1.00E-06	117 kb downstream of *SHH*
	155723112	kgp11597900	C	T	1.00E-06	118 kb downstream of *SHH*

**Table 2:** Pairwise statistics of *LMBR1* and *SHH*

	patient-patient			normal-normal			patient-normal	
Gene	No. patient	No.IBD in patient pairs	% IBD in patient pairs	No. normal	No.IBD in normal pairs	% IBD in normal pairs	No.IBD in patient-normal pairs	% IBD in patient-normal pairs
*LMBR1*	21	84	0.400	22	17	0.074	29	0.126
*SHH*	21	81	0.386	22	16	0.069	24	0.104

% IBD patient pairs = IBD patient pairs/case x (case-1)/2

% IBD normal pairs = IBD normal pairs/normal x (normal-1)/2

% IBD patient-normal pairs = IBD patient-normal pairs/case x normal/2

## Discussion

Our IBD analyses refine the mapping of the candidate regions for PPD into two ~380 Kb segments in 7q36 referring to *LMBR1* and *SHH* genes, respectively (Additional file 3: Table S2). The segment for *LMBR1* includes three genes (i.e. *LMBR1*, *NOM1*, and *RNF32*) and lies within the 450 kb candidate region identified before [6].Mutations in the ZRS is playing an important role in the pathogenesis of PPD (Additional file 4: Table S3). The duplication of ZRS can cause TPTPS and SD4 [18]. Its role in PPD-II /PPD-III is unclear. In the previous investigation of the same family, Li et al. detected no pathogenic mutation in ZRS as well as no duplication of ZRS [19]. Consequently, the etiology of this PPD family may be another limb-specific regulatory element of *SHH* gene exists in the noncoding region.

In addition to the segment of *LMBR1*, we also identified a segment of *SHH*. The *SHH* gene encodes sonic hedgehog, a secreted protein, which plays a key role in the limb development [28]. The ectopic expression of *SHH* in the anterior limb margin can cause PPD in human, in mouse [29], Hemingway cat [7] and chicken [30]. Recently, Petit et al. identified a 2 kb deletion occurring about 240 kb upstream from the *SHH* promoter in a large family with PPD-hypertrichosis. They found the 2 kb deletion repress the transcriptional activity of the *SHH* promoter in vitro [31]. It raises a possibility that long range regulation may be an explanation for the PPD. Further target-enrichment sequencing and further functional experiments for *LMBR1* and *SHH* are required to identify the pathogenic mutation(s).

In summary, we refine the mapping of the candidate regions for PPD based on high-density genomic SNPs. The potential candidate mutations are most likely to locate in *LMBR1* and/or *SHH* gene. It is much improved compred with previous results [6, 19]. Our study suggests that the IBD approach is a suitable method for mapping the causal genes of human diseases. Moreover, as disruptions of topological chromatin domains can result in limb malformations [32], more attention should be paid when studying PPD in the future on this aspect.

## Additional files

Additional file 1: Figure S1. Plot of the distribution of the IBD segments.

Additional file 2: Table S1. Top 0.01% peak signals.

Additional file 3: Table S2. IBD segments of *LMBR1* and *SHH*.

Additional file 4: Table S3. Mutations in intron 5 of *LMBR1.*

## Abbreviations

IBD: Identity by descent
*LINC00244*: Long intergenic non-protein coding RNA 244
*LMBR1*: Limb development membrane protein 1
*MNX1*: Motor neuron and pancreas homeobox 1
*NOM1*: Nucleolar protein with MIF4G domain 1
PPD: Preaxial polydactyly
*RNF32*: Ring finger protein 32
