# Supplementary figures and images for: Identity-by-descent refines mapping of candidate regions for preaxial polydactyly II /III in a large Chinese pedigree
Source: Hereditas. 2017 Jul 3;155:2. doi: 10.1186/s41065-017-0040-6 (PMC5496229; doi:10.1186/s41065-017-0040-6)

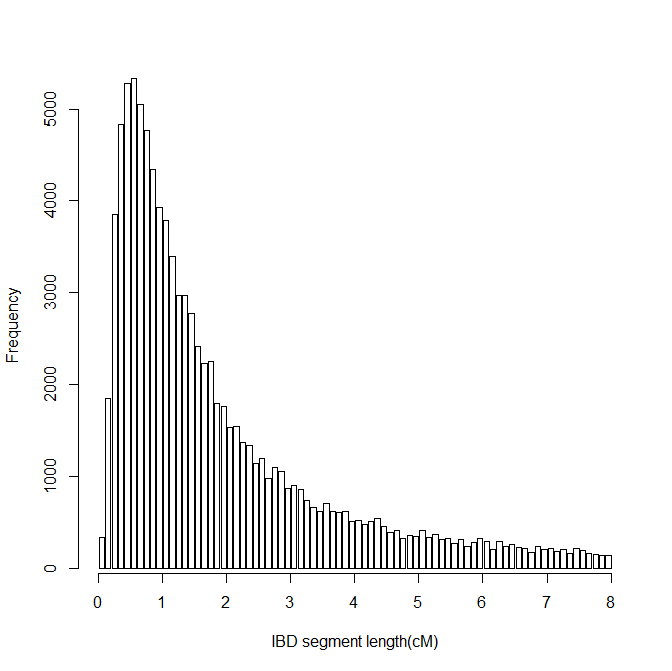

Supplement: Supplementary file 1 — Plot of the distribution of the IBD segments. [file 41065_2017_40_MOESM1_ESM.tiff]
